# Prognosis in Pulmonary Tuberculosis

**Published:** 1909-06-19

**Authors:** J. Edward Squire

**Affiliations:** Senior Physician Mount Vernon Hospital for Diseases of the Chest; Consulting Physician, Sanatorium for Workers, Benenden


### Hospital Clinics.

### PROGNOSIS IN PULMONARY TUBERCULOSIS.

By J. EDWARD SQUIRE, C.B., M.D. (Lond.), F.R.C.P.; Senior Physician Mount Vernon Hospital for Diseases of the Chest; Consulting Physician, Sanatorium for Workers, Benenden.

With the advance in our knowledge of the pathology of tuberculous processes and more rational methods in the treatment of consumptives, the questions to be answered in giving a prognosis in pulmonary tuberculosis involve much more than an estimate of the time within which a fatal termination may be expected to occur.

Within the memory of many of us the certainty of a fatal result of the disease was taken for granted; all that was asked was "How long do you think the patient will last?" Fortunately, we have learned that tuberculosis, even when it attacks the lungs, is a disease which is often arrested, and that complete recovery is by no means uncommon. The records of autopsies on persons dying from diseases other than tuberculosis have shown how large a proportion of the people in this country become affected and recover with nothing beyond a scar in the lung to mark the old seat of the disease, and clinical experience provides full endorsement of the deductions from pathological evidence. Not only do we sometimes meet with evidences in the lungs of healthy persons of scars—and perhaps on inquiry learn of a period of indefinite ill-health extending over many months which has almost been forgotten—but we may watch the consumptive recovering under our eyes, and test the permanency of the cure by following the record of years of healthy useful life in the former patient.

A fresh question has now assumed primary importance in the prognosis of consumption—namely, what are the patient's chances of recovery? The matter of prognosis in pulmonary tuberculosis has become one of considerable complexity involving many considerations; it includes not merely a forecast of the ultimate result—the probability of recovery—but an estimate of the probable duration of the illness, whether the result be favourable or otherwise. If we may hope for arrest of the disease, will recovery be complete, allowing active work and the power to earn a livelihood, or will it merely mean a life of dependence and partial invalidism? Further, if there is apparently complete recovery, what are the chances of a relapse in the years to come? In all these questions, so vitally important to the patient, of such urgent interest to his friends, we are asked to form some estimate of duration. Will he get well? How long will it take? Will he be able to get back to work? Is there any chance of his getting another attack? How long before he may consider himself practically safe from a relapse? These questions come tumbling out one after the other, and others of a similar nature, referring to the special circumstances of each individual patient, often follow in rapid succession. Truly the man who is called upon to give a prognosis in a case of pulmonary tuberculosis has a difficult task to perform and a great responsibility to undertake.

There is no rule which will apply to all cases, no formula which can be employed to satisfy all inquirers—each case must be considered in connection with all the circumstances, and an opinion reasoned out for each individual patient. In coming to a decision we must take into consideration the present condition of the patient, the extent and degree of activity of the disease processes, the resisting power of the individual. We must consider the type of the disease, the length of time during which it has been in progress; and the condition of other organs, especially in respect to the presence of tuberculosis. We must also take into account the age, sex, and physique of the patient, his family history, the conditions under which he is living (pecuniary as well as hygienic), the nature of his occupation, and the kind of employment he will take up after treatment.

Of all these various points which must be examined and weighed there is probably not one which is of such general and such essential importance in prognosis as the *stage of the disease* in the lungs. The curability of consumption bears a very direct relation to the stage of the disease at which systematic treatment is commenced. Not only is the expectation of cure well justified in early cases, but the time necessary to effect a cure will be much shorter if treatment is commenced in an early stage than when the disease has become more advanced.

With every advance of the disease the expectation of cure diminishes, the length of time required to bring about arrest of the disease increases, and the ultimate result in the event of arrest becomes less satisfactory. This is now well known to all who have studied the treatment of consumptives, and is evidenced in all reports of sanatoria in this and other countries. It should therefore be the aim of every medical practitioner to ensure the early recognition of tuberculosis in the lungs with the object of commencing systematic treatment without delay.

In regard to Prognosis, what do we understand by an Early Case? Naturally, the length of time which has elapsed since the earliest symptoms—that is, the duration of the disease—is one of the factors to be considered. It is, however, not the only one, since the disease-processes advance with different degrees of rapidity in different cases; so that two persons becoming infected at the same time may be at very different stages of the disease at the end of a month or two. Nor can we define a certain stage of the disease-process in any part of the lung as being our measure of an early case; for in one case the disease may advance quickly to softening in a restricted area of the lung, whilst in another it spreads throughout the organ without showing great activity in any part. With the element of time we must also consider the extent of lung involved as well as the stage of development of the disease-process in the area of most advanced mischief.

The most satisfactory condition is where, with a short history, a restricted area of one lobe is alone involved and the mischief has not advanced beyond consolidation. It is in sending the patient for advice whilst in this condition that an attack of hæmoptysis, coming without any previous suspicion of lung trouble as the first warning or symptom of illness, is so important.

The significance of the amount of lung involved as a guide to prognosis is well illustrated by the following table, which is compiled from the reports of the Mount Vernon Hospital.

Table Showing relation of Improvement to Number of Lobes Affected in 2,720 Cases of Pulmonary Tuberculosis.

| Lobes Affected | Cases | Much Improved | Improved | Total Improved |
|---|---|---|---|---|
| 1 | 877 | 512 (58.38 %) | 251 (28.62 %) | 763 (87.00 %) |
| 2 | 1,015 | 384 (37.83 %) | 352 (34.67 %) | 736 (72.50 %) |
| 3 | 515 | 116 (22.52 %) | 183 (35.53 %) | 299 (58.05 %) |
| 4 | 277 | 42 (15.16 %) | 81 (29.24 %) | 123 (44.40 %) |

When we examine the cases in which actual arrest of the disease has occurred, the figures are even more significant, as the following abstract from the Report for 1908 of the Northwood branch of the Mount Vernon Hospital shows:—

"Of 55 cases in which the disease was arrested, 49—or 89 per cent.—were patients with only one lobe affected. The remainder had two lobes affected. The disease could not be considered arrested in any of the patients with 3, 4, or 5 lobes affected."

The *activity of the disease*, as shown by the general condition of the patient and the temperature record, is of some importance, but must be considered in relation to the stage of the disease. The temperature should always be taken into account in prognosis, examining its height, daily range, and the tendency to be readily disturbed. The rapidity of the pulse should also be noted, and also whether it is easily quickened. Instability of pulse and temperature are significant of activity of the disease and of small resisting power.

In later stages of the mischief raised temperature and quickened pulse have less significance in themselves in prognosis, since these signs are to be expected as the disease extends. Of greater importance as a guide to prognosis when the disease has advanced sufficiently is the *type which the illness shows in its development*. Broadly speaking, we may recognise three well-defined types: First, there is the acute limited infection, where the process, though active, is confined to a limited area in which the disease expends itself, consolidation being rapidly followed by softening and excavation. Such cases, though perhaps very acute, with high temperature and possibly great general disturbance, are often very satisfactory in their complete recovery, especially if the softening quickly opens into a fair-sized tube through which the *débris* may be expelled.

The second type is sub-acute: in this the disease spreads over a wider area, but never reaches any great activity in any part; it extends itself in all directions, instead of concentrating its activity on one spot. This type is very unsatisfactory; its progress is comparatively slow, but its advance cannot be checked, or at least is difficult to check, so that though the illness is not so marked and the temperature rarely so high as in the first type, the hope of ultimate arrest is less, the time required to bring about arrest is longer, and the ultimate result, when arrest is brought about, less satisfactory.

The third type—less common than either of the others, except in persons beyond middle age, in chronic bronchitics, or in alcoholics—is chronic in its course, leads to fibroid overgrowth in the lung, and though its progress is slow it does not tend to arrest. Life may be prolonged for years, but it is the burdensome life of the chronic invalid. This classification is by no means exhaustive; for example, there are the so-called pneumonic cases, where a considerable extent of lung may be involved and the disease-processes develop rapidly. Nor are the types always well defined; but many cases in which we are called upon to forecast the future will fall into one or other of the three classes sketched above.

Having determined the probable tendency of the type of disease present, we must endeavour to ascertain how this may be modified by the extent of *resisting power in the individual*. The young and robust country labourer who has become accidentally infected will be able to overcome an acute and active tuberculosis, which would be much more serious in the weedy indoor worker of a large city, and which would probably prove rapidly fatal to the flabby victim of dissipation. In assessing the amount of resisting power, we must take into consideration the hereditary tendency to suffer from tuberculosis as shown by the family history, the physique and "condition" of the patient, the shape and expansion of the chest, the condition of the circulatory organs, the digestive powers, etc. The mode of life, condition of the home, occupation, and habits all have a bearing on the question, as do also such details as the state of the finances and other matters which may cause anxiety or worry to the patient. It is not uncommon to find that a patient, who is doing less well than had been anticipated, is worried over the expenses of his treatment or the difficulties in which his family is placed by his prolonged absence from work. Many a man, whose chances of recovery seemed most favourable, has had to throw away his chance because of the necessity of returning to work before the disease was completely arrested.

In gauging the resisting power in any individual there is much which only knowledge of men and experience of illness can teach. Temperament cannot be entirely left out of account. There are those who seem determined to get well and appear almost to overcome the disease by their pluck and fighting qualities; others give up the fight before it has well commenced and need constant encouragement to make them offer even a poor resistance. A type familiar to most of us—neurotic, excitable, and easily upset—offers poor resistance to illness.

In reference to occupation, we must sometimes consider the kind of employment which the patient will take up when he returns to work, for this may have an important bearing on the permanence of his cure.

After leaving a sanatorium, or after a term of systematic treatment at home, when the disease has become arrested it is usually necessary to exercise care for a considerable time in order to ensure that the disease does not light up again into renewed activity. The nature of the employment is frequently of less importance than the conditions under which it is carried out. Of all unhealthy occupations, one of the most injurious is that—whatever its nature—which does not bring in a living wage. It is often better to return to an occupation which in itself is unsuitable, but which is well paid, than to take up a light and healthy job which will not bring in enough to live on. For this reason there is much to be said in favour of a discharged patient returning to his old occupation.

*The age* of the patient has an important bearing on prognosis. In young children the outlook is always grave, and the younger the child the more serious is the danger. Yet the large number of cases of children dying from other causes in which scars of old and healed tuberculosis are found in the lungs shows that recovery from pulmonary tuberculosis frequently takes place. From the age of ten until puberty the mortality from pulmonary tuberculosis again rises, especially amongst girls.

Early adult life (18 to 30 years) seems to be the most favourable to recovery; after about 30 or 35 years of age recovery appears to be slower, and after 45 years the chances of ultimate cure are comparatively small, though the disease may become quiescent and life may drag on for several years. Many middle-aged consumptives so far recover as to be able to work, with occasional lapses when they have to give up for a time. This may go on for several years, during which they are able to earn a living, and thus an " economic cure " may be attained; but the disease is not actually cured, and occasionally lights up into activity until the " chronic consumptive " becomes a total invalid. In patients of 50 and over cure is hardly to be anticipated, though the progress of the disease is usually slow.

*Sex* has some bearing upon prognosis, especially during the years of sexual activity. Girls at about the age of puberty frequently show little resisting power against tuberculosis, though I have noticed that if the disease commences at this period girls in whom menstruation has not yet occurred seem to have a better chance of recovery than those in whom the menstrual periods have become established. It may be, as I have suggested, that a reserve of energy stored up at this period, if not already expended in the establishment of functional development, may be utilised in combating the disease. During the child-bearing period we have to take into account the debilitating effects of frequent pregnancies and of over-prolonged lactation. Women whose strength has been sapped by these causes are unable to offer adequate resistance to an attack of tuberculosis. When consumption attacks a woman during pregnancy, or when a consumptive woman becomes pregnant, the disease frequently remains more or less quiescent; after confinement, however, the disease often becomes active and advances rapidly.

Sex has an influence on the nature of the employment and the conditions of life generally. The married woman who becomes consumptive is freed from some of the anxiety as to ways and means which worries the male consumptive on whose work the family relies for a livelihood. The woman engaged in domestic duties can attend the hospital for advice without the risk of losing her employment, and as a result we find that a larger proportion of women than of men are admitted into hospitals or sanatoria in the early stages of the complaint. The men cannot afford to give up work, either to seek advice (when the nature of the complaint may be recognised early), or to enter the sanatorium for treatment—until the disease is so advanced that work is almost impossible. Women are said to recover from pulmonary tuberculosis more frequently than men. If this be so it is probably to be explained by the larger proportion of women who come under treatment before the disease has become advanced. In the Northwood Report (1909) it is stated that in 27.9 per cent. of the males and 46.5 per cent. of the females only one lobe was affected. As the result of treatment the disease was arrested in 9.1 per cent. of all the male patients, and in 18.2 per cent. of all the females. The male cases admitted generally presented much more extensive disease than the females.

When a patient has been under observation for some time, and the course of the disease has been watched, there are further data by which we may be guided in our prognosis. The temperature records for several weeks show clearly the severity of the toxæmia, and possibly will give some indication of the recuperative power of the patient. A temperature which remains continuously above the normal shows an unfavourable case, as does also the oscillating temperature with wide diurnal range. Both of these types go with severe toxæmia.

A persistently subnormal temperature may indicate small resisting power, though it must be borne in mind that under open-air conditions, especially in the colder weather, the patient's temperatures are frequently below the normal for many weeks. The progress towards recovery is often marked on the temperature chart by three periods. At first the temperature is persistently above the normal, gradually falling to below 98°, where it remains with slight oscillations for a few weeks, and then, as strength is regained, rising to the normal line and varying only about half a degree on either side of this.

The diminution in pulse-rate and increase in weight are also indications of improvement, though too much reliance should not be placed on the increase in weight. The presence or absence of bacilli in the sputum gives little or no assistance in prognosis, and the number of bacilli and the variations in their number at different examinations are of no significance in this direction. Excavation and evacuation are often important stages in the curative process, but will be accompanied by increase—and possibly by very considerable increase — in the number of bacilli found in the sputum. Some prognostic indications may be obtained by repeated observations on the opsonic index, but these are uncertain and unreliable except in the hands of experts in this method of examination.

*Complications* may seriously affect the prognosis in pulmonary tuberculosis. Tuberculosis in other organs not only taxes the general resisting power of the body, but may be an indication that resistance has been overcome and is inadequate. Phthisis supervening in an individual who is already the subject of a tuberculosis affecting bones or joints is unlikely to take a favourable course; whilst on the other hand the infection of other organs—larynx, intestines, kidneys, liver, etc.—in a phthisical patient makes the prognosis additionally grave. Albuminuria and persistent diarrhœa are often signs of the beginning of the end.

Laryngeal tuberculosis is an unfavourable complication. Life may be prolonged for several years even when the larynx has become infected in the course of pulmonary tuberculosis, and cure apparently occurs in certain cases. When the larynx is affected at the commencement of a pulmonary tuberculosis, the lung and the larynx being attacked almost simultaneously, the prognosis is, in my experience, very unfavourable, and the course is generally acute and often short.

Complications independent of tuberculosis are often met with in cases of consumption. Heart disease, if it does not predispose to tuberculosis, adds to the seriousness of the illness; when at all advanced, especially when compensation has failed and the heart is dilated, it is a very unfavourable complication. Bronchitis is a serious addition to pulmonary tuberculosis; bronchiectasis adds much to the discomforts of the disease and to the risks of secondary (septic) infections. Pleurisy is of slight importance in prognosis unless there is considerable effusion; if the effusion is purulent, necessitating operation, there is an added danger. Intercurrent diseases—acute illnesses coming on in the course of pulmonary tuberculosis—may check improvement, and may light up the mischief into activity, causing it to advance with a rapidity and severity greater than at any previous stage of the illness. On the other hand, we see consumptives pass through a sharp attack of influenza or other illness with no ultimate ill-effects.

Since tuberculosis progresses comparatively slowly, and accidental complications may at any time occur, there is always the chance that our prognostications, however carefully reasoned out, may prove to be unrealised. Hæmoptysis or meningitis, pneumothorax or some other unforeseen accident may change the whole course of the illness.

I was once called in consultation to a case of consumption in a lady of 50, in order to advise as to the movements of a daughter who was abroad, and whom the mother was anxious to see. It was a case in which everything pointed to a chronic course, and in the ordinary way it seemed that any time within the next six months would do for the daughter's return. Some chance remark by the nurse about the patient's condition during the preceding night made me think of meningitis, and I fortunately communicated my suspicions to the medical attendant, and advised sending to the daughter without delay. The patient died within a week or ten days. It does not do to be too dogmatic in giving a prognosis in pulmonary tuberculosis. The most favourable cases sometimes disappoint us, and, on the other hand, some of the apparently hopeless cases occasionally get well. I have seen cases of active tuberculosis in the lungs which showed no signs of improvement for six months suddenly begin to improve and ultimately recover. After all, we are asked to gauge from experience of similar cases the probable course of events; we ought not to be expected to prophesy.

We may be misled in the ultimate result; we must, however, expect to be proved wrong if we attempt to fix a time at which the result, favourable or otherwise, may be expected. The very expression of an opinion may lead to an alteration of conditions which have a material influence on the event. The expectation that the patient will live but a few weeks longer perhaps leads to such additional attention and solicitude that his life is prolonged for months—as, for example, when a poor patient is removed to the infirmary or to a home for the dying. On the other hand, we express the opinion that a hospital patient may live several months, and he is in consequence taken home, to die in a few days under the altered conditions.

This consideration suggests another element which must be taken into account in making a prognosis, namely the conditions under which the patient is placed, and the facilities for systematic treatment. Immediate removal of a patient to a good sanatorium may make all the difference to his chances of recovery; delay in commencing systematic treatment may destroy his chance completely. It is this which makes the lamentably inadequate accommodation for the sanatorium treatment of poor consumptives such a serious national misfortune.

I have said that in the matter of time, our estimates must frequently be at fault. It is well, however, to bear in mind that only in quite early cases can we anticipate cure in so short a time as three months. The activity of the disease may in more advanced cases be arrested in about three months; but further care will be required for many months, and complete cure may be a matter of years. When, however, arrest has been brought about, the individual may follow his employment, though he should remain under occasional medical supervision. Many "unsatisfactory results of sanatorium treatment" are due to the want of appreciation of the difference between quiescence and cure, which has led to the premature relinquishing of necessary precautions and consequent relapse.

Though not, strictly speaking, involved in the medical aspect of prognosis in consumption, we should bear in mind the possible moral effect of prolonged idleness—or at least of rest in comparative comfort—of persons of slight strength of will. It is found that amongst the working-class patients there is a danger that the long stay in a sanatorium which is often necessary for cure results in a distaste for work, so that when discharged physically fit for work they have lost all desire to work—a moral deterioration which materially diminishes their usefulness as wage-earners. It is in counteracting or overcoming this that labour colonies for cured (or arrested) consumptives would probably prove of the greatest value.

